# microRNA-374a suppresses colon cancer progression by directly reducing CCND1 to inactivate the PI3K/AKT pathway

**DOI:** 10.18632/oncotarget.9320

**Published:** 2016-05-12

**Authors:** Yiyu Chen, Jingwen Jiang, Mengyang Zhao, Xiaojun Luo, Zixi Liang, Yan Zhen, Qiaofen Fu, Xiaojie Deng, Xian Lin, Libo Li, Rongcheng Luo, Zhen Liu, Weiyi Fang

**Affiliations:** ^1^ Cancer Center, Traditional Chinese Medicine-Integrated Hospital of Southern Medical University, Guangzhou, PR China; ^2^ Cancer Research Institute, Southern Medical University, Guangzhou, PR China; ^3^ Department of Pathology, School of Basic Medicine, Guangzhou Medical College, Guangzhou, PR China

**Keywords:** miR-374a, CCND1, CRC, PI3K/AKT pathway, EMT

## Abstract

microRNA-374a (miR-374a) exhibits oncogenic functions in various tumor types. Here we report that miR-374a suppresses proliferation, invasion, migration and intrahepatic metastasis in colon adenocarcinoma cell lines HCT116 and SW620. Notably, we detected that PI3K/AKT signaling and its downstream cell cycle factors including c-Myc, cyclin D1 (CCND1), CDK4 and epithelial-mesenchymal transition (EMT)-related genes including ZEB1, N-cadherin, Vimentin, Slug, and Snail were all significantly downregulated after miR-374a overexpression. Conversely, cell cycle inhibitors p21 and p27 were upregulated. Expression of E-cadherin was only decreased in HCT116, without any obvious differences observed in SW620 cells. Furthermore, luciferase reporter assays confirmed that miR-374a could directly reduce CCND1. Interestingly, when CCND1 was silenced or overexpressed, levels of pPI3K, pAkt as well as cell cycle and EMT genes were respectively downregulated or upregulated. We examined miR-374a levels by *in situ* hybridization and its correlation with CCND1 expression in CRC tumor tissues. High miR-374a expression with low level of CCND1 was protective factor in CRC. Together these findings indicate that miR-374a inactivates the PI3K/AKT axis by inhibiting CCND1, suppressing of colon cancer progression.

## INTRODUCTION

Colorectal cancer (CRC) is one of the most common malignant gastrointestinal carcinomas with a rising morbidity and mortality. In recent studies, microRNA deregulations have been frequently observed and induce the pathogenesis of human colorectal cancers [1–6]. miR-374a, located on chromosome Xq13.2, promotes or suppresses development of human cancer according to different studies. Some reports identified that miR-374a triggers epithelial-mesenchymal transition (EMT) in breast cancer and promotes cell proliferation in gastric cancer [7, 8], while Võsa et al. found low expression of miR-374a in early-stage NSCLC is correlated with poorer patient survival [9]. In colorectal cancer, Slattery et al. found that hsa-miR-374a-5p significantly reduces mortality for all 1141 CRC cases examined [10]. Xu et al. confirmed miR-374a in colon tumor is significantly downregulated compared with paired normal tissue [11].

Here we had a deeper investigation into miR-374a biological functions and its possible signaling mechanisms in colon cancer. Unlike previous reports in other cancers [7, 8], we showed miR-374a considerably inhibits proliferation, invasion and migration both *in vitro* and *in vivo*. We found that miR-374a inactives PI3K/AKT pathway by directly reducing CCND1, a link not previously published to our knowledge. Further, we analyzed the negative correlation between miR-374a and CCND1 expression in colon cancer patients, finding this pattern was closely related with prognosis. Our data suggest that miR-374a functions as a tumor suppressor in CRC.

## RESULTS

### miR-374a overexpresssion inhibits proliferation, invasion, migration, and intrahepatic metastasis of CRC cells *in vitro* and *in vivo*

**Figure 1:**
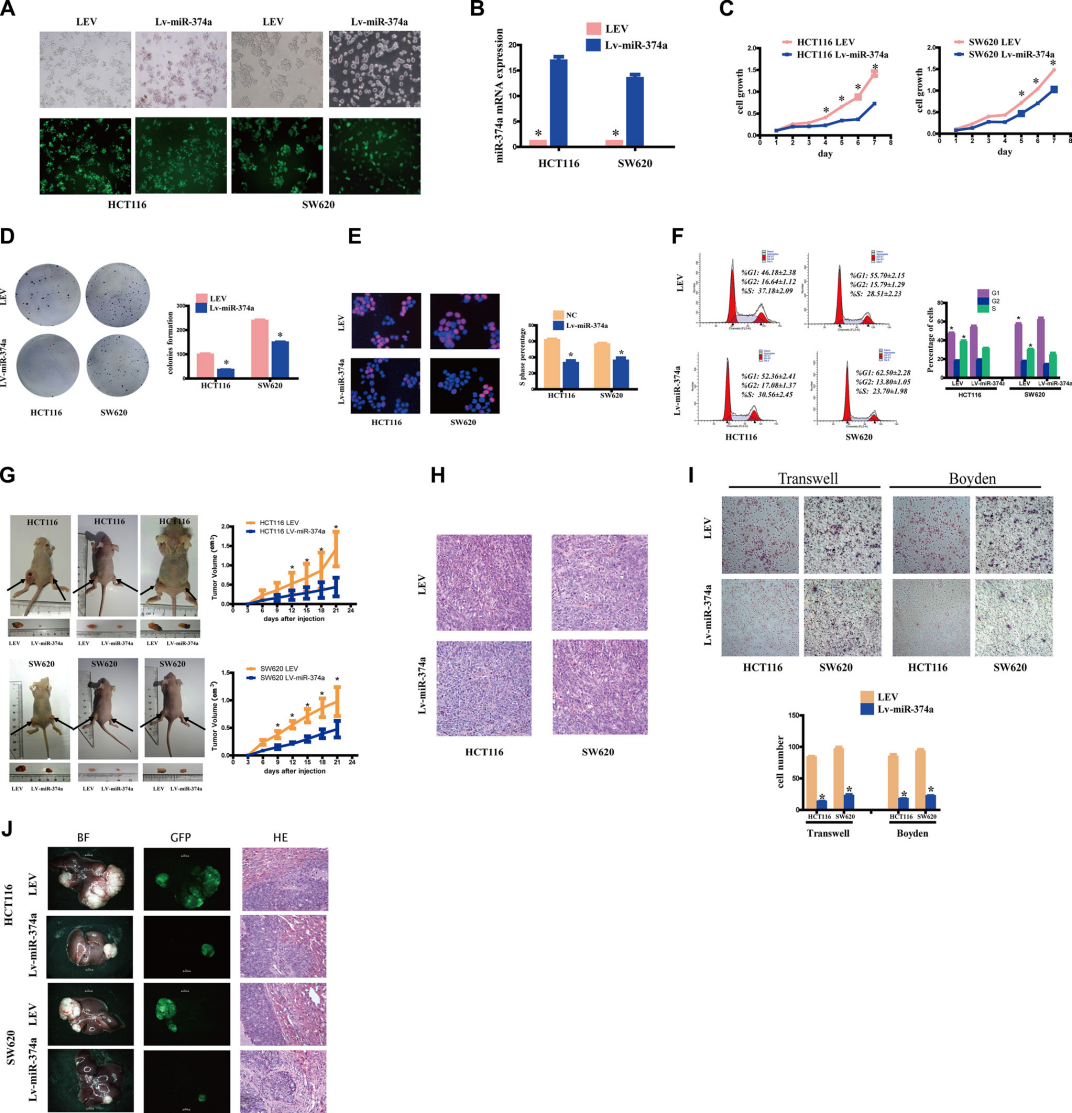
miR-374a overexpresssion inhibits proliferation, invasion and migration *in vitro* and *in vivo* (**A**) HCT116 and SW620 cells were transfected with Lv-miR-374a and LEV. Green fluorescent protein (GFP) expression was used to monitor the transfection efficiency. (**B**) miR-374a expression measured by qRT-PCR following overexpression of Lv-miR-374a. Experiments were repeated three times with similar results, and error bars represent mean ± SEM, *P < 0.05. C-F. The *in vitro* function of miR-374a was measured by MTT assay (**C**), Colony-forming assay (**D**), Edu assays (**E**), FCM (**F**) in HCT116-Lv-miR-374a and SW620-Lv-miR-374a cells. Experiments were repeated three times with similar results, and error bars represent mean ± SEM, *P < 0.05. G-H. Excised tumors 20 days after HCT116-Lv-miR-374a and SW620-Lv-miR-374a implantation (**G**) and representative H&E staining (**H**) of primary cancer tissues are shown. Black arrows showed the tumors. (**I**) Effect on invasion and migration of miR-374a was measured by Transwell and Boyden Chamber assays in HCT116-Lv-miR-374a and SW620-Lv-miR-374a cells. Experiments were repeated three times with similar results, and error bars represent mean ± SEM, *P < 0.05. (**J**) *In vivo* intrahepatic metastasis assays results after HCT116-Lv-miR-374a and SW620-Lv-miR-374a injection.

To test the biological functions of miR-374a *in vitro*, we infected colorectal cancer cell lines HCT116 and SW620 with lentivirus carrying miR-374a sequence, established HCT116-Lv-miR-374a and SW620-Lv-miR-374a stable cell lines (Figure 1A). Quantitative real-time PCR (qRT-PCR) confirmed the increased expression of miR-374a in these lines, which were 10–20 folds higher than those of control lentiviral empty vector (LEV)cells (Figure 1B).

HCT116-Lv-miR-374a cells exhibited decreased proliferation within 72 hr, and the difference was still statistically significant until day7 (Figure 1C). Colony formation assays also supported this growth inhibition (Figure 1D). Further, Edu assay (Figure 1E) and flow cytometry (FCM) (Figure 1F) showed that miR-374a suppressed cell cycle transition from G1 to S phase.

To investigate whether the effects of miR-374a translated *in vivo*, we subcutaneously injected cells into nude mice. The xenograft results in HCT116-Lv-miR-374a and SW620-Lv-miR-374a cells were markedly smaller and lighter than corresponding control cells (Figure 1G). HE images were showed in Figure 1H.

For invasion and migration analysis, HCT116-Lv-miR-374a, SW620-Lv-miR-374a, or control parental lines were cultured in Transwell or Boyden Chambers. Consistent with growth assays mentioned above, we found that miR-374a-overexpressing lines had reduced invasive and migratory ability compared to controls (Figure 1I). *In vivo* intrahepatic metastasis assay showed HCT116-Lv-miR-374a and SW620-Lv-miR-374a had reduced intrahepatic metastasis ability versus controls (Figure 1J).

**Figure 2:**
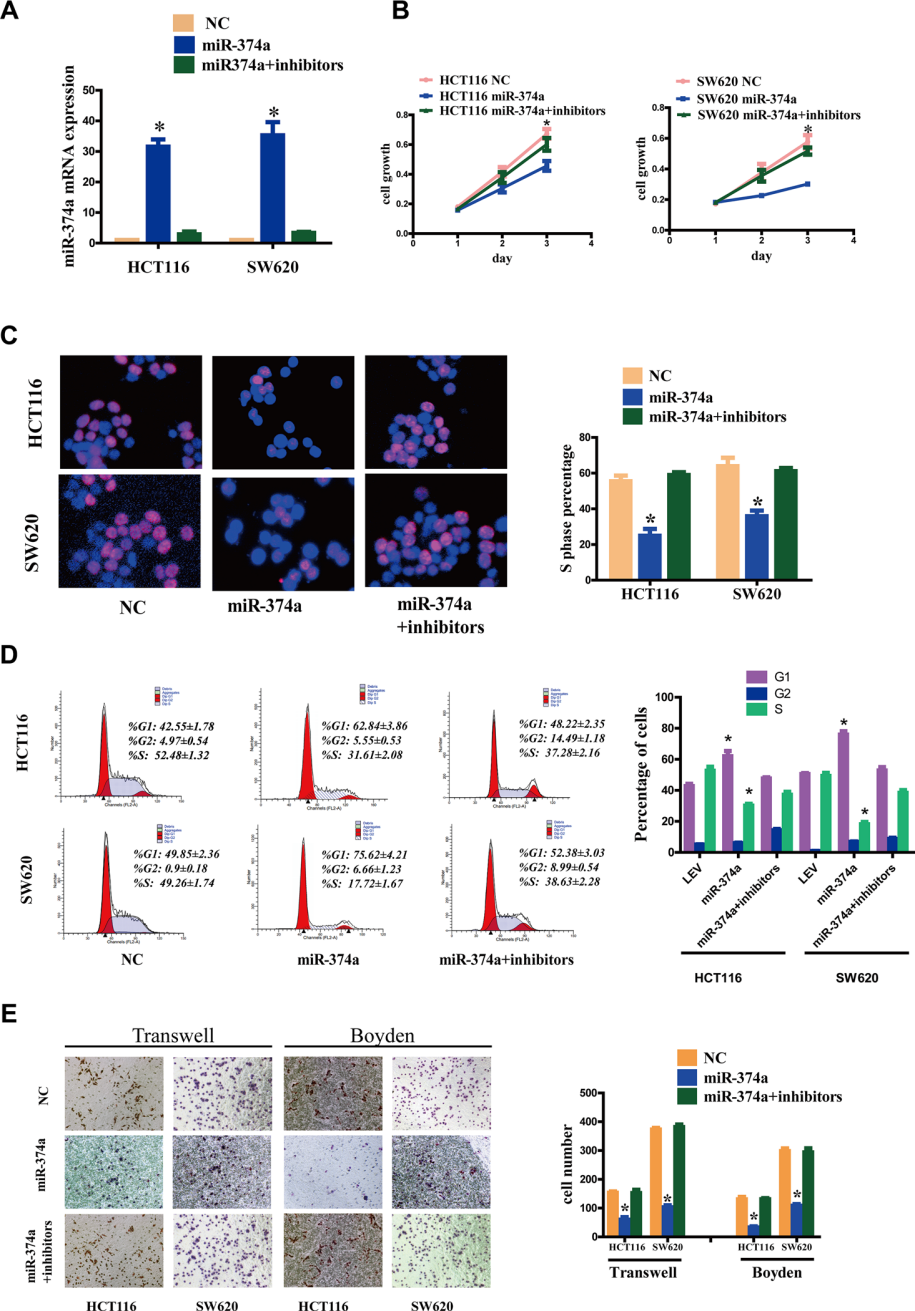
miR-374a inhibition rescues the suppressive functions on proliferation, invasion and migration (**A**) HCT116-Lv-miR-374a and SW620-Lv-miR-374a cells were transfected with miR-374a inhibitors or controls. miR-374a expression measured by qRT-PCR following. Experiments were repeated three times with similar results, and error bars represent mean ± SEM,*P < 0.05. B-E. Cell viability was measured at selected time points (**B**). Cell cycle was measured by Edu assays (**C**) and FCM (**D**) respectively. Invasion and migration was measured by Transwell and Boyden Chamber assays (**E**). Experiments were repeated three times with similar results, and error bars represent mean ± SEM, *P < 0.05.

To confirm the specificity of these effects, we transfected HCT116-Lv-miR-374a and SW620-Lv-miR-374a with miR-374a inhibitors or controls (Figure 2A). Using MTT (Figure 2B), Edu (Figure 2C), FCM (Figure 2D), Transwell and Boyden Chamber (Figure 2E) assays, we validated the suppressive function of miR-374a.

### miR-374a inactives PI3K/AKT signaling and downstream ce ll cycle, EMT factors

**Figure 3:**
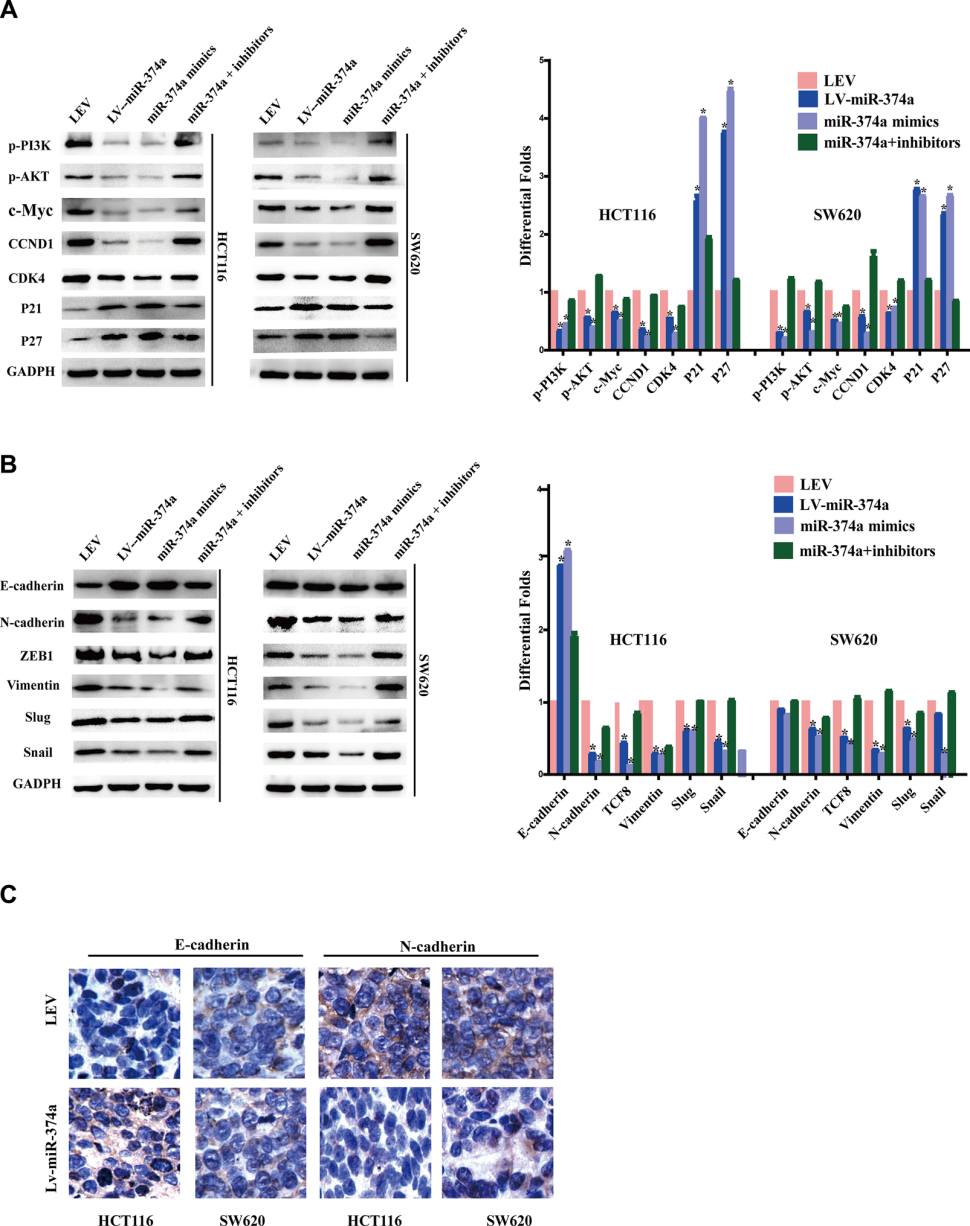
miR-374a is a negative regulator of PI3K/AKT signaling and suppresses cell cycle, invasion and migration relevant genes (**A**–**B**) Western blot analysis of the protein levels of p-PI3K, p-AKT, c-Myc, CCND1, CDK4, P21, P27 (A) as well as invasion and migration relevant protein levels of E-cadherin, N-cadherin, ZEB1, Vimentin, Slug and Snail (B) in response to overexpressed or silenced miR-374a expression. (**C**) Expression of E-cadherin and N-cadherin in tumor tissues were measured by IHC.

In order to define a CRC-specific pathway that miR-374a regulates, we designed four groups, LEV, LV-miR-374a, miR-374a mimics, miR-374a+inhibitors. Using western blot, we found that miR-374a not only suppressed levels of *phos*-PI3K (Tyr458) and *phos*-Akt (Ser473), but also significantly decreased the expression of cell cycle-related genes including c-Myc, CCND1, and CDK4. In addition, p21 and p27 were upregulated after miR-374a overexpression (Figure 3A).

Based on invasion and migration assay results, we examined expression of EMT markers including N-cadherin, ZEB1, Vimentin, Slug, and Snail, finding they were all downregulated by miR-374a. E-cadherin was upregulated, but only in HCT116-Lv-miR-374a, whereas no changes were observed in SW620-Lv-miR-374a cells (Figure 3B). Further, IHC analysis confirmed upregulated E-cadherin expression in miR-374a-overexpressed xenograft group (no any obvious differences observed in SW620 cells), and downregulated N-cadherin expression compared with negative control (Figure 3C).

### miR-374a directly binds to the CCND1 3′UTR

**Figure 4:**
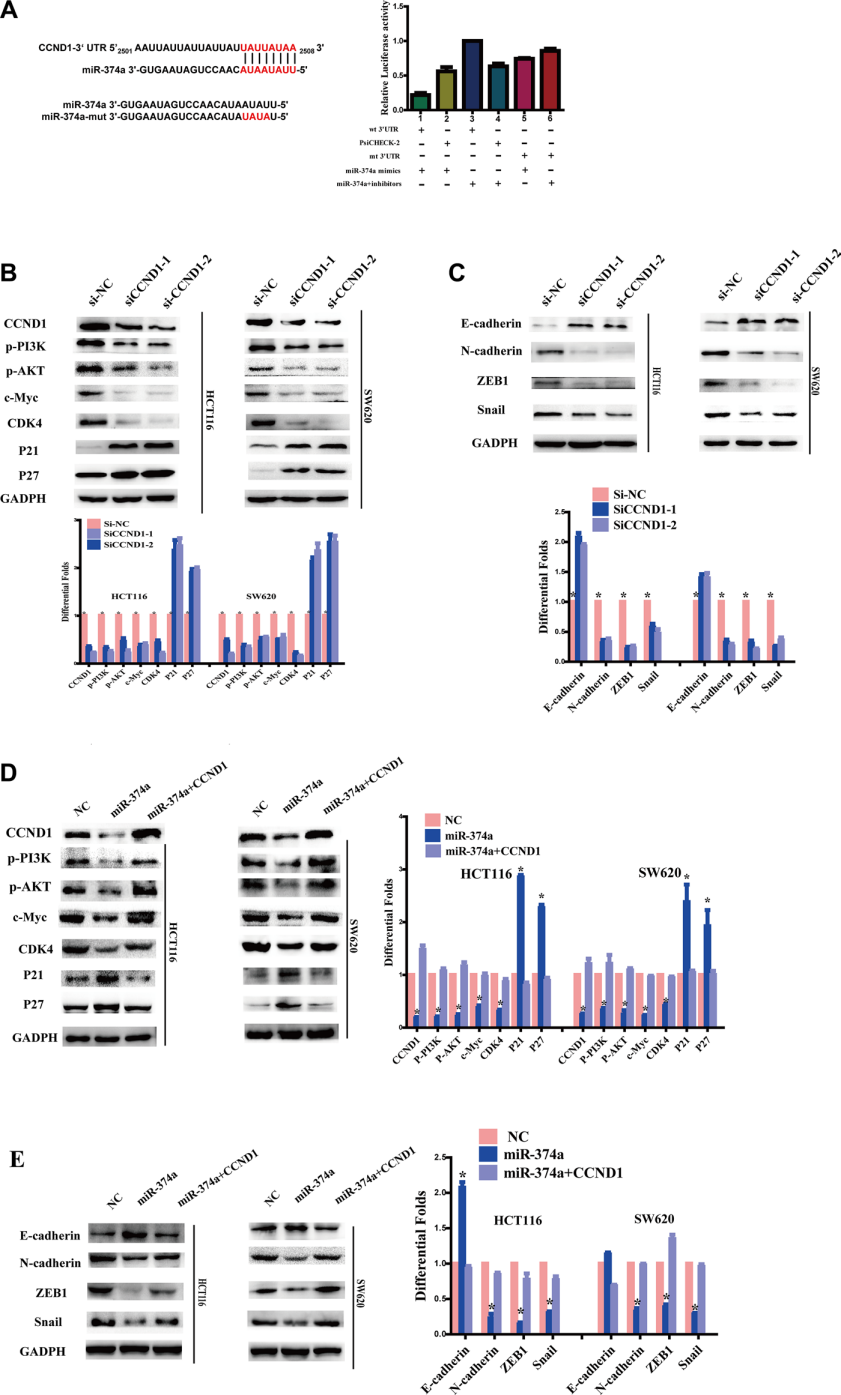
As a target of miR-374a, CCND1 exerts feedback on PI3K/AKT pathway and downstream cell cycle and EMT genes (**A**) miR-374a and its putative binding sequences in the 3′UTR of CCND1. Mutations were generated in the complementary site that binds to miR-374a. Luciferase reporter assays were used to determine whether miR-374a directly binds the 3′UTR of CCND1. Experiments were repeated three times with similar results, and error bars represent mean ± SEM, *P < 0.05. (**B**–**C**) HCT116 and SW620 cells were transfected with two sequences of si-CCND1 or controls. Western blot analyzed the expression of CCND1, p-PI3K, p-AKT, c-Myc, CDK4, P21, P27 (B) as well as invasion and migration relevant protein levels of E-cadherin, N-cadherin, ZEB1, Snail (C). (**D**–**E**) HCT116-Lv-miR-374a and SW620-Lv-miR-374a cells were transfected with a plasmid encoding CCND1. Western blot analyzed the expression of CCND1, p-PI3K, p-AKT, c-Myc, CDK4, P21, P27 (D) as well as invasion and migration relevant protein levels of E-cadherin, N-cadherin, ZEB1, Snail (E).

TargetScan (http://www.targetscan.org/) and PicTar (http://pictar.mdc-berlin.de/) bioinformatics algorithms were used to explore the specific target of miR-374a. We found that miR-374a was predicted to bind to the CCND1 3′UTR. Wild-type (Wt) or mutant (mt) 3′UTR vectors for CCND1 were cotransfected together with miR-374a mimics or inhibitors into 293T cells. Luciferase activity linked with the CCND1 3′UTR was suppressed in a dose-dependent manner in miR-374a mimic-transfected 293T cells versus controls. Conversely, inhibition of miR-374a resulted in a significant increase in luciferase reporter activity controlled by the CCND1 3′UTR (Figure 4A). Mutating the miR-374a sequence reversed its suppressive functions. Previous results demonstrated miR-374a overexpression led to reduced CCND1 expression. After miR-374a inhibitors were transfected into HCT116-Lv-miR-374a and SW620-Lv-miR-374a cells, CCND1 levels were restored. Taken together, these data suggest that miR-374a directly reduces CCND1.

### CCND1 exerts feedback on the PI3K/AKT axis

To study whether miR-374a suppresses PI3K/AKT pathway via directly reducing CCND1 in colon cancer, we transfected HCT116 and SW620 cells with two sequences of si-CCND1 or control. Western blots confirmed CCND1 was effectively silenced by siRNA (Figure 4B). MTT assays (Figure S1A) and Edu assays (Figure S1B) also supported its knockdown. Of note, levels of p-PI3K and p-AKT were notably reduced as expected, as well as c-Myc and CDK4 (Figure 4B). Inversely, cell cycle inhibitors P21 and P27 expression were elevated accordingly.

CCND1 interference also had a great impact on invasion and migration. Transwell and Boyden Chamber assays validated that reduced CCND1 expression suppressed cell invasion and migration (Figure S1C). Western blots showed the expression of N-cadherin, ZEB1, and Snail all decreased while E-cadherin levels increased. (Figure 4C)

**Figure 5:**
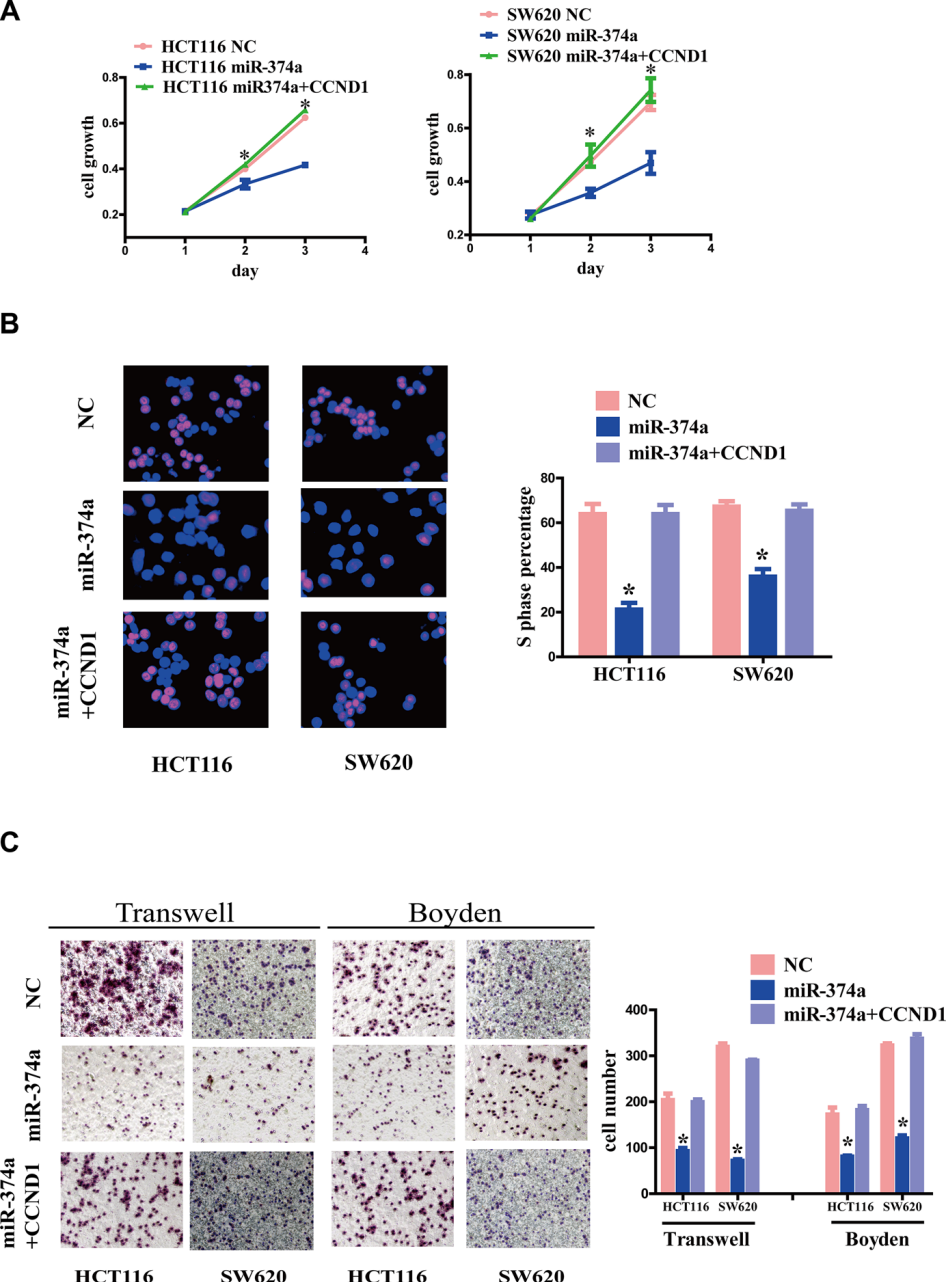
The effect of CCND1 re-overexpression on proliferation, invasion and migration (**A**–**C**) HCT116-Lv-miR-374a and SW620-Lv-miR-374a cells were transfected with a plasmid encoding CCND1. Cell growth and cell cycle were measured by MTT assays (A) and Edu assays (B). Invasion and migration was measured by Transwell and Boyden Chamber assays (C). Experiments were repeated three times with similar results, and error bars represent mean ± SEM, *P < 0.05.

On the contrary, when we re-overexpressed CCND1 in HCT116-Lv-miR-374a and SW620-Lv-miR-374a cells, levels of p-PI3K, p-AKT and EMT-related factors were rescued (Figure 4D–4E). *In vitro* assays, including MTT assay (Figure 5A), Edu assay (Figure 5B), Transwell and Boyden Chamber assays (Figure 5C) showed the same results as western blot.

### Correlation between miR-374a and CCND1 in colon cancer tissues and their clinical significance

**Figure 6:**
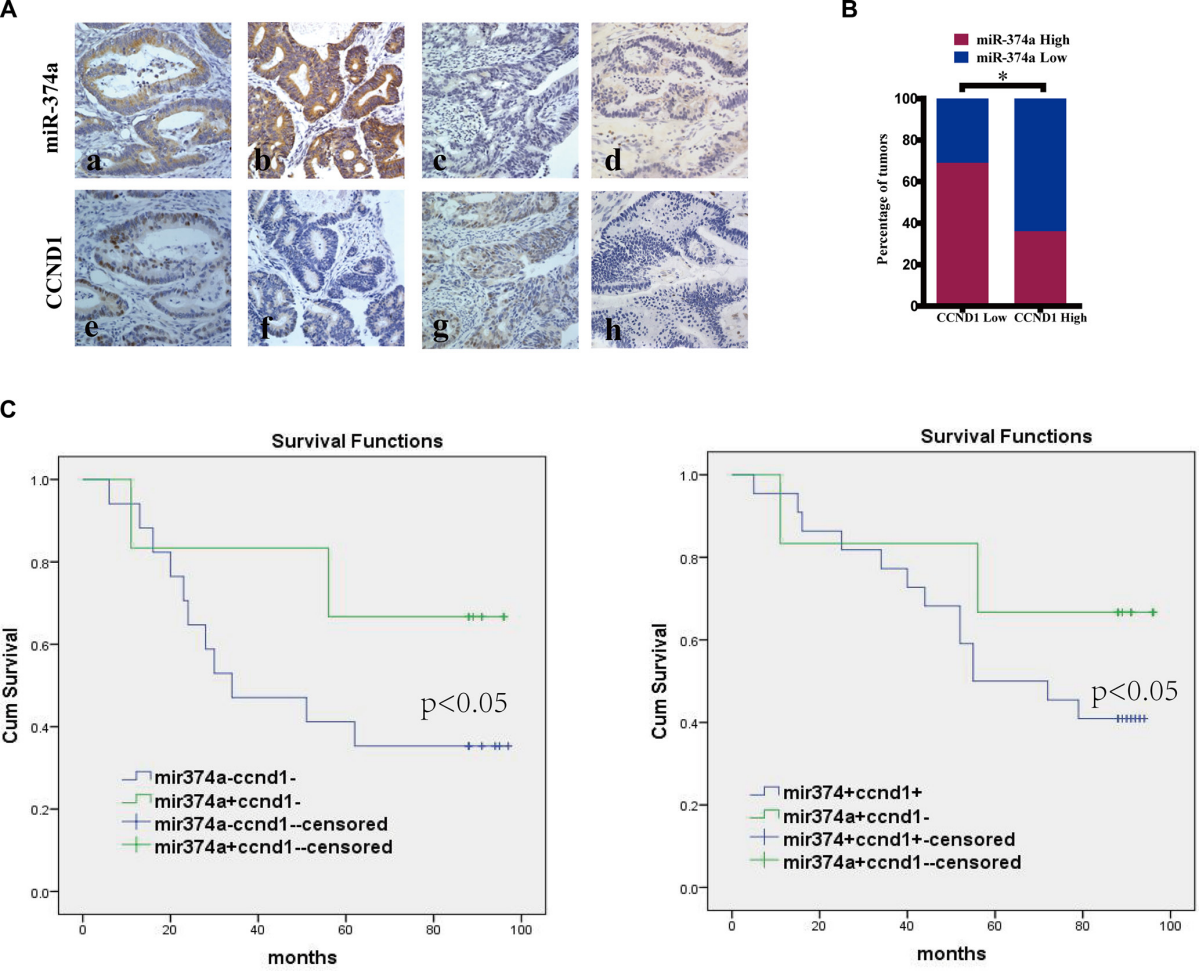
The correlation between miR-374a and CCND1 in colon cancer tissues and their clinical significance (**A**) *In situ* hybridization and immunohistochemistry results showing the expression level of miR-374a and CCND1 at same locations in colon cancer tissues. Figures a, b, e, g represent high expression, c, d, f, and h represent low expression. Among them, figure a and e were form case1, b and f from case2, c and g from case 3, d and h from case 4. Original magnification 400×. (**B**) Percentage of specimens exhibit low or high miR-374a expression in relation to CCND1 expression. (**C**) Kaplan-Meier survival curve comparing subgroups based on miR374a or CCND1 expression in the primary tumors of colon cancer patients: low CCND1 expression cases with either high or low miR-374a expression as well as high miR-374a expression cases with either high or low CCND1 expression. *P* value is based on a log-rank test.

We next examined the expression of miR-374a and CCND1 in 90 colon tumor tissues by *in situ* hybridization (ISH) and immunohistochemistry (IHC) respectively (Figure 6A). The clinical parameters and prognostic value for miR-374a and CCND1 in colorectal cancer were analyzed respectively (Table S1, Table S2 Figure S2). We found no significant correlation between miR-374a expression and clinical parameters including age, gender, T stage, N stage and pathological grading. Kaplan-Meier analysis showed miR-374a expression had no significant influence on overall survival. For CCND1, we found the CCND1 expression was statistically correlated with T stage (no significant correlation with other factors) and low levels of CCND1 had a better overall survival than high ones.

The relationship between miR-374a and CCND1 was then examined by correlation analysis (Figure 6B). Samples were divided into four groups: miR-374a high expression / CCND1 high expression (HH), miR-374a high expression / CCND1 low expression (HL), miR-374a low expression / CCND1 high expression (LH), miR-374a low expression / CCND1 low expression (LL). When investigating the relationship between miR-374a and CCND1 expression with clinical features and prognosis, we found there were no significant correlations for factors including age, gender, T stage, N stage and pathological grading (Table 1). Kaplan-Meier analysis showed patients with high miR-374a expression /low CCND1 expression had better prognoses than low miR-374a expression/low CCND1 expression ones. Within the subgroup of patients with high miR-374a expression levels, those with low CCND1 expression lived longer than those with high CCND1 expression (Figure 6C). The summary of univariate and multivariate Cox regression analyses of overall survival were described in Table 2, where we found N stage was an independent prognosis factor (*P* < 0.05). Patients with N0 status had a longer survival time compared to those with N1–N2 status. Collectively, we defined high miR-374a expression with low CCND1 expression as positive prognostic factor.

**Table 1:** Correlation between the clinical pathological factors and expression of miR-374a, CCND1 in CRC

The expression of miR-374a and CCND1							
Clinical parameter	*n*	HH (*n*, %)	HL (*n*, %)	LH (*n*, %)	LL (*n*, %)	*χ*^2^ value	*P* value
gender							
male	48	11 (22.9)	6 (12.5)	20 (41.7)	11 (22.9)	4.218	0.239
female	42	12 (28.6)	1 (2.4)	22 (52.4)	7 (16.7)		
age (year)							
> 65	52	13 (25)	5 (9.6)	24 (46.2)	10 (19.2)	10.028	0.018
≤ 65	38	10 (26.3)	2 (5.3)	18 (47.4)	8 (21.1)		
T stage							
T_1_–T_2_	15	2 (13.3)	1 (6.7)	9 (60.0)	3 (20.0)	1.766	0.622
T_3_–T_4_	75	21 (28.0)	6 (8.0)	33 (44.0)	15 (20.0)		
N stage							
N_0_	58	13 (22.4)	6 (10.3)	30 (51.7)	9 (15.5)	4.545	0.208
N_1_–N_2_	32	10 (31.2)	1 (3.1)	12 (37.5)	9 (28.1)		
pathological grading							
I–II	48	13 (27.1)	4 (8.3)	24 (50.0)	7 (14.6)	1.889	0.596
III–IV	42	10 (23.8)	3 (7.1)	18 (42.9)	11 (26.2)		

* HH represents high miR-374a with high CCND1 expression, HL represents high miR-374a with low CCND1 expression, LH represents low miR-374a with high CCND1 expression, LL represents low miR-374a with low CCND1 expression.

**Table 2:** Summary of univariate and multivariate Cox regression analysis of overall survival duration

Parameter	Univariate analysis			Multivariate analysis		
	*P*	HR	95% CI	*P*	HR	95% CI
Age						
> 65 years vs ≤ 65	0.723	0.922	0.589–1.444			
Gender						
Male vs. Female	0.977	0.992	0.581–1.695			
FIGO stage						
I–II vs. II–III+III	0.18	1.442	0.845–2.461			
T classification						
T1–T2 vs. T3–T4	0.234	1.621	0.732–3.590			
N classification						
N0 vs. N1 + N2	0.003	2.289	1.332–3.934	0.011	2.037	1.176 3.527
miR-374a/CCND1 expression						
HH vs HL vs LH vs LL	0.265	1.157	0.896–1.494			

* HH represents high miR-374a with high CCND1 expression, HL represents high miR-374a with low CCND1 expression, LH represents low miR-374a with high CCND1 expression, LL represents low miR-374a with low CCND1 expression.

## DISCUSSION

Unlike previous reports [7, 8], we demonstrated that overexpression of miR-374a results in cell cycle arrest, reduced cell invasion, migration and intrahepatic metastasis in *in vitro* and *in vivo* colon cancer models. The reverse effects were observed after inhibition of miR-374a. Together these data, miR-374a functions as a tumor suppressor in colon cancer.

The PI3K/AKT pathway promotes tumor cell proliferation by inducing cell cycle transition signal [12–14]. In our study, levels of p-PI3K, p-AKT, c-Myc, CCND1, CDK4 were all significantly decreased while p21 and p27 were upregulated after miR-374a overexpresssion. Our findings suggested that miR-374a inactives the PI3K/AKT pathway and its downstream c-Myc-stimulated cell cycle pathway, thereby inhibiting cell proliferation in colon cancer.

It is well known that PI3K/AKT pathway can also promotes EMT signal transition [15–18]. In our study, N-cadherin, ZEB1, Vimentin, Slug and Snail were all markedly decreased by miR-374a. The expression of E-cadherin increased in HCT116-Lv-miR-374a but not in SW620-Lv-miR-374a cells, which is likely attributed to differences between the two cell lines. Our data revealed that miR-374a suppressed EMT genes by suppressing the PI3K/AKT pathway to inhibit invasion and migration. Based on above studies, PI3K/AKT signaling is involved in the inhibitory effect of miR-374a on cell proliferation, invasion and migration in colon cancer. However, the precise molecular mechanism of miR-374a modulates PI3K/AKT signaling remains to be determined.

To further understand the role of miR-374a, the target gene of miR-374a was identified. We previously reported CCND1 was suppressed by miR-374a in colon cancer. Interestingly, this protein was predicted as a direct target of miR-374a by bioinformatics software analysis. Subsequently, we confirmed this prediction through luciferase reporter assays and found CCND1 could reverse the inhibitory effects of miR-374a. Our data demonstrates that miR-374a functions as a tumor suppressor by directly reducing CCND1, in contrast with previous reports that miR-374a acts as oncogene by modulating tumor suppressors WIFI, PTEN and WNT5A [7].

Amplification and overexpression of CCND1, which alters cell cycle progression, is frequently observed in a variety of tumors [19]. CCND1 has recently been associated with cell adhesion and motility in primary bone macrophages [20]. Decreased cyclin D1 and cyclin D1-CDK4/6 kinase activity reduces invasion and migration in breast cancer cells [21]. Interestingly, when we silenced or overexpressed CCND1, PI3K/AKT pathway and its downstream cell cycle and EMT factors were respectively suppressed or stimulated. These results indicate that CCND1 reverses the inhibition induced by miR-374a to activate PI3K/AKT signaling in colon cancer, a result which has not been reported before.

Previous reports found that miR-374a reduced mortality in CRC cases [10] and high levels of CCND1 were associated with poor prognosis in colon cancer [22]. However, the correlation between miR-374a and CCND1 expression in CRC has not been investigated before. In this study, we found that there was a significantly negative correlation between miR-374a and CCND1 expression. Though pathological parameters were not correlated with miR-374a levels, survival analysis implied that patients with high miR-374a expression and low CCND1 expression had longer overall survival times than other three groups, supporting that miR-374a acts as a tumor suppressor in CRC.

**Figure 7:**
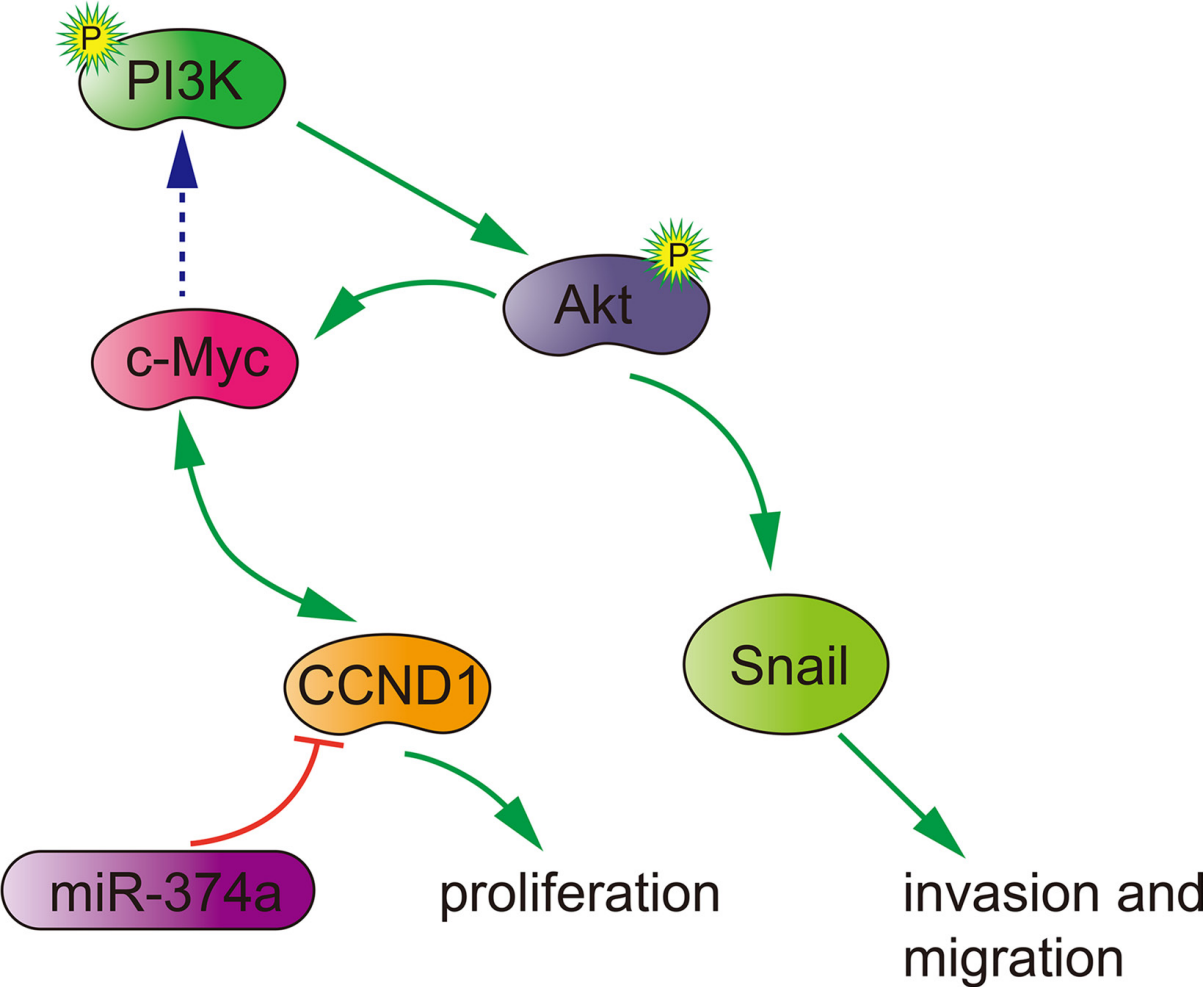
Potential signaling pathway utilized by miR-374a to suppress proliferation, invasion and migration in colon cancer

In summary, miR-374a functions as a tumor suppressor by inactivating PI3K/AKT signaling and downstream signals. This negative regulation is accomplishing by directly reducing CCND1 to inhibit proliferation, invasion and migration in colon cancer cells (Figure 7). We also observed that high miR-374a and low CCND1 expression in patient samples is associated with favorable outcomes, which suggests that miR-374a and CCND1 may be useful prognostic biomarkers. Because targeting driver pathways represents the best option to tailor cancer treatment and improve survival in patients [23], our study indicates that overexpressing miR-374a may be a promising therapy for colorectal cancer patients.

## MATERIALS AND METHODS

### Cell culture and tissue specimens

Purchased from ATCC Bioresource Center, HCT116 (ATCC-CCL-247) cell line was obtained from a male adult's colonic carcinoma surgery through the auspices of the DAB Tissue Procurement Service in 1981 [24]. SW620 cell line was purchased from Chinese Academy of Sciences Cell Bank (Shanghai, China). The parental cell line was isolated from the tissue of a 51-year-old Caucasian male and initiated by A. Leibovitz, et al. This line was derived from a metastasis lymph node from SW480 [25]. HCT116 was cultured in RPMI 1640 medium (HyClone, Logan, UT) supplemented with 10% FBS (ExCell, Shanghai, China). SW620 was cultured in Dulbecco's modified Eagle's medium (DMEM, HyClone, Logan, UT) supplemented with 10% fetal bovine serum (FBS) (ExCell, Shanghai, China). Both cell lines were grown in a humidified chamber with 5% CO_2_ at 37°C. Tissue microarrays with 90 colon cancer samples were purchased from Shanghai Outdo Biotech Co., Itd., China.

### Lentivirus production and infection

Lentiviral particles carrying hsa-miR-374a precursor and its flanking control sequence were constructed by GeneChem, Shanghai, China. HCT116 and SW620 cells were infected with lentiviral vector, and polyclonal cells with green fluorescent protein signals were selected for further experiments using fluorescence-activated cell sorting flow cytometry. Total RNA from these cell clones was isolated, and levels of miR-374a were quantified by qRT-PCR.

### RNA isolation, reverse transcription, and qRT-PCR

RNA isolation, reverse transcription, and qRT-PCR were performed in colon cancer cell lines according to a previous description [26]. Specific sense primers of miR-374a, U6, CCND1 and ARF5 are shown in Supplementary Tables 3 and 4.

### Transient transfection with siRNAs, plasmid or miR-374a mimics/inhibitors

SiRNAs and miR-374a mimics and its inhibitors were designed and synthesized by RiboBio Inc. (Guangzhou, China). The sequences of miR-374a mimics, inhibitor and their respective controls are shown in Supplementary Table 5. The CCND1 plasmid was purchased from Biosense (Guangzhou, China). Twenty-four hours before transfection, colon cancer cells HCT116 and SW620 were seeded onto a 6- or 96-well plates (Nest, Biotech, China) at 30–50% confluence. CCND1 siRNA, plasmid and miRNAs mimics, inhibitors were then transfected at a working concentration of 100 nm using TurboFect siRNA Transfection Reagent (Fermentas, Vilnius, Lithuania) according to the manufacturer's protocol. Cells were collected after 48–72 hr. The sequences of CCND1 siRNA were shown in Supplementary Table 6.

### Western blot analysis

Western blot analysis was performed according to a previous description [27]. Antibodies included anti-CCND1, CDK4, CDK6, p21, p27, c-Myc, p-AKT (Ser473), p-PI3K (Tyr458), ZEB1, E-cadherin, N-cadherin, Vimentin, Slug, Snail (1:1000; Cell Signaling Technology, Danvers, MA, USA) and GADPH antibody (1:1000; CW Biotechnology). Images were captured with ChemiDocTM CRS+ Molecular Imager (Bio-Rad).

### Cell proliferation analysis

Cell proliferation was analyzed by MTT assay according to a previous description [28]. Experiments were performed three times.

### Colony formation assay

Colony formation assay was analyzed according to a previous description [29]. Experiments were performed three times.

### Cell cycle analysis, edu incorporation assays

Cell cycle analysis, Edu incorporation assay were performed according to a previous description [27]. Each experiment was performed in triplicate.

### *In vitro* cell invasion and migration assays

*In vitro* cell invasion and migration assays were performed according to a previous description [30]. Each experiment was performed in triplicate.

### *In vivo* tumorigenesis and metastasis assays in nude mice

For *in vivo* tumorigenesis assays, a total of 1 × 10^7^ logarithmically growing HCT116 or SW620 cells overexpressing miR-374a and their control cells in 0.1 ml RPMI 1640 /DMEM medium were respectively subcutaneously injected into the left or right flank of 4–6-week-old male BALB/c nu/nu mice (*N* = 3).

For *in vivo* metastasis assay, 50 μl of HCT116 and SW620 cells (1 × 10^7^) overexpressing miR-374a or their control cells were injected under the liver capsule of each mouse (3 mice for each group), and then carefully pushed its liver back into the abdominal cavity after cleaning and lightly pressing the pinhole with alcohol cotton balls for 2 min. All mice were killed in 6 weeks. Their liver organs were subjected to fluorescent image detection using LT-9MACIMSYSPLUS whole-body imaging system (Lighttools Research, Encintas, CA, USA). The mice were maintained in a barrier facility on HEPA-filtered racks and fed with an autoclaved laboratory rodent diet. All animal studies were conducted in accordance with the principles and procedures outlined in Southern Medical University Guide for the Care and Use of Animals.

### *In situ* hybridization

*In situ* hybridization was performed on formalin-fixed paraffin-embedded sections (4 μm thickness) of tumor specimens. After processing with 3% H_2_O_2_, sections were treated with proteinase K (2 μg/ml) at 37°C for 30 min, washed, and prehybridized for 2 h at 37°C. Hybridization with digoxygenin (DIG)-labeled miRCURY LNA probes (probe sense: 5′-cACTTATCAGGTTGTATTATAa -3′; Exiqon, Woburn, MA, USA) was performed overnight at37°C. Slides were then washed at 37°C and incubated with alkaline phosphatase–conjugated sheep anti-DIG Fab fragments for 1h at room temperature. Staining was visualized by adding BM purple AP substrate (Roche, Basel, Switzerland) according to the manufacturer's instructions.

### Immunohistochemical staining

Paraffin sections prepared from *in vivo* experiments were used for immunohistochemistry to detect protein expression levels of E-cadherin, N-cadherin and CCND1. The indirect streptavidin-peroxidase method was utilized based on the manufacturer's instructions. Stained tissue sections were examined separately by two pathologists. The antibodies used were rabbit anti-E-cadherin (1:50, proteintech), rabbit anti- N-cadherin (1:50, proteintech), rabbit anti-CCND1 (1: 250, Abcam) respectively.

### Evaluation of staining

The *in situ* hybridized and immunohistochemically stained tissue sections were reviewed and scored separately by two pathologists blinded to clinical parameters. For cytoplasm staining, the score was evaluated according to the sum of cytoplasm staining intensity and the percentage of positive staining cells. For nuclear staining, The staining intensity was scored as 0 (negative), 1 (weak), 2 (medium) and 3 (strong) [31] and the percentage of positive staining cells was defined in a scale of 0–3 (0: < 1%, 1: 1–9%, 2: 10–50% and 3: > 50%). For nuclear staining, the score was defined based on the sum of nuclear staining intensity and the number of positive nuclear staining. The positive nuclear staining scores were defined as follows: 0: < 20%, 1:20–49%, 2: 50–79% and 3: > 80%. Expression levels of miR-374a, E-cadherin, and N-cadherin were evaluated by cytoplasm staining evaluation, a sum of final staining score 0–4 or 5–6 were considered to be low or high-expression levels, respectively. For CCND1, the sum of the cytoplasm and nuclear staining scores were used as the final staining scores (0–12). For statistical analysis, a final staining score of 0–6 or 7–12 were considered to be low or high-expression levels, respectively.

### MicroRNA target validation

CCND1 was predicted to be a direct target of miR-374a by TargetScan (Prediction of microRNA targets) software. Fragments (452-bp or 350-bp) of the CCND1 3′UTR were amplified by PCR primers (Supplementary Table 7) and cloned into psiCHECK-2 vectors (named wt). Site-directed mutagenesis of the miR-374a binding site in the CCND1 3′UTR was performed using GeneTailor Site-Directed Mutagenesis System (Invitrogen; named mt). For reporter assays, wild-type (wt), mutant (mt) or control psiCHECK-2 vector were cotransfected into HCT116 and SW620 cells together with miR-374a mimics or inhibitors. Luciferase activity was measured at 48 hr after transfection by Dual-Luciferase Reporter Assay System (Promega Corporation, Madison, WI, USA).

### Statistical analysis

All quantified data represented an average of at least triplicate samples. IBM SPSS v20.0 (IBM Corporation, Armonk, NY, USA) and GraphPad Prism v5.0 (GraphPad Software, Inc., La Jolla, CA, USA) software were used for statistical analysis. Data are presented as mean ± SEM. One-way analysis of variance or two-tailed Student's *t*-test was used for comparisons between groups. Fischer's or χ^2^-test was used to identify differences between categorical variables. Partial correlations were applied in multivariate correlations analyses. Survival analysis was performed by Kaplan–Meier method. Differences were considered statistically significant when *P* < 0.05.

## SUPPLEMENTARY FIGURES AND TABLES


