# Supplementary material for: microRNA-374a suppresses colon cancer progression by directly reducing CCND1 to inactivate the PI3K/AKT pathway
Source: Oncotarget. 2016 May 12;7(27):41306–19. doi: 10.18632/oncotarget.9320 (PMC5173061; doi:10.18632/oncotarget.9320)
Supplement: Supplementary file 1 [file oncotarget-07-41306-s001.pdf]

microRNA-374a suppresses colon cancer progression by directly reducing CCND1 to inactivate the PI3K/AKT pathway

Supplementary Materials

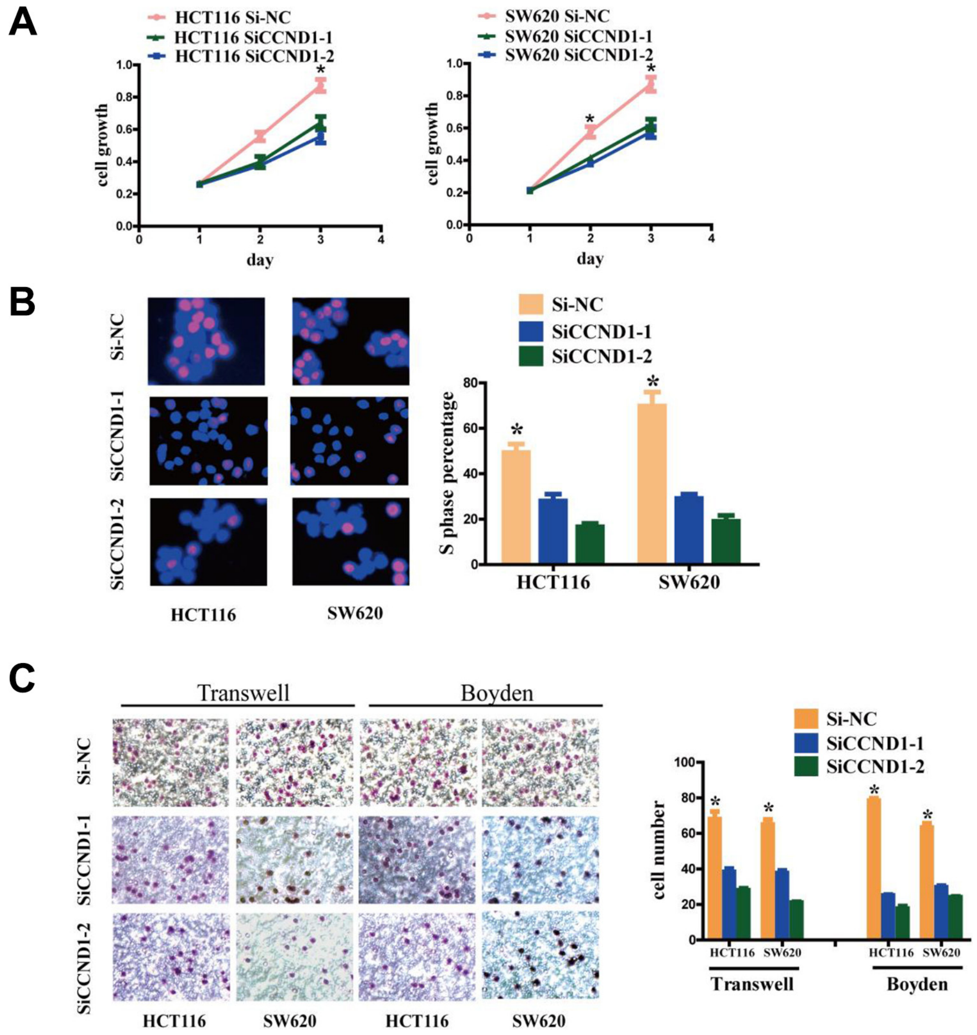

Supplementary Figure S1: The effect of CCND1 silencing on proliferation, invasion and migration. (**A**–**C**) HCT116 and SW620 cells were transfected with two sequences of si-CCND1. Cell growth and cell cycle were measured by MTT assays (A) and Edu assays (B). Invasion and migration were measured by Transwell and Boyden Chamber assays (C).

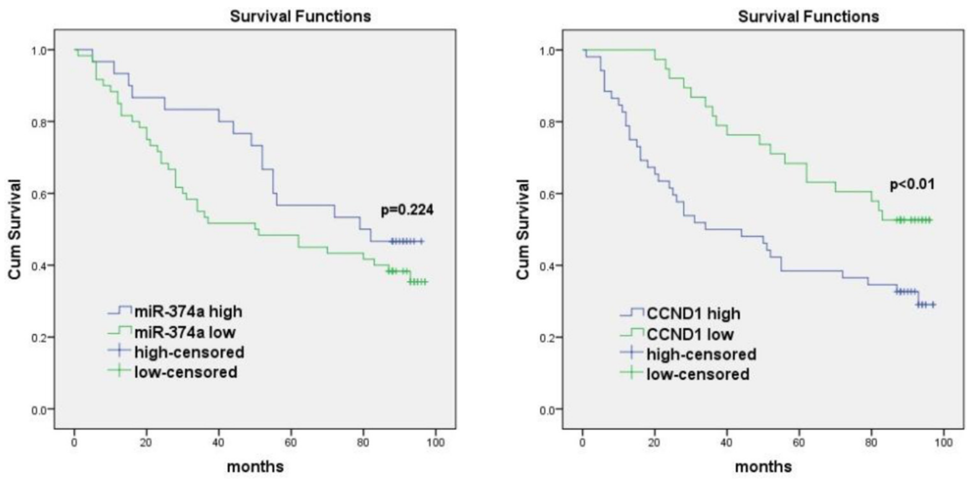

Supplementary Figure S2: Respective survival curve of miR-374a expression (left) and CCND1 expression (right) in CRC tissues.

Supplementary Table S1: Correlation between the clinical pathological factors and expression of miR-374a in CRC specimens

Clinical parameter		*n*	High expression (*n* %)	Low expression (*n* %)	χ^2 value	*P* value
gender						
male	48	17 (35.4)	31 (64.6)	0.201	0.654	
female	42	13 (31)	29 (69)			
age (year)						
> 65	52	18 (34,6)	34 (65.4)	0.091	0.763	
≤ 65	38	12 (31.6)	26 (68.4)			
T stage						
T1–T2	15	3 (20)	12 (80)	1.44	0.23	
T3–T4	75	27 (36)	48 (64)			
N stage						
N_0	58	19 (32.8)	39 (67.2)	0.024	0.876	
N1–N2	32	11 (34.4)	21(65.6)			
Grade						
I–II	48	18 (37.5)	30 (62.5)	0.804	0.37	
III–IV	42	12 (28.6)	30 (71.4)			

Supplementary Table S2: Correlation between the clinical pathological factors and expression of CCND1 in CRC specimens

Clinical parameter		*n*	High expression (*n* %)	Low expression (*n* %)	χ^2 value	*P* value
gender						
male	48	24 (50)	24 (50)	2.551	0.110	
female	42	28 (66.7)	14 (33.3)			
age (year)						
> 65	52	31 (59.6)	21 (40.4)	0.170	0.680	
≤ 65	38	21 (55.3)	17 (44.7)			
T stage						
T_1–T_2	15	3 (20)	12 (80)	10.53	0.001	
T_3–T_4	75	49 (65.3)	26 (34.7)			
N stage						
N_0	58	30 (51.7)	28 (48.3)	2.450	0.117	
N_1–N_2	32	22 (68.8)	10(31.2)			
Grade						
I–II	48	29 (60.4)	19 (39.6)	0.294	0.588	
III–IV	42	23 (54.8)	19 (45.2)			

Supplementary Table S3: Sequences of miR-374a and U6

		Sequence
miR-374a	Sense	5′ CGGCGGTTATAATACAACCTG3′
	Antisense	----
U6	Sense	5′ CTCGCTTCGGCAGCACATATA3′
	Antisense	----

Supplementary Table S4: Primer sequences for CCND1 and ARF5

		Sequence
CCND1	Sense	5′GACGGCCGAGAAGCTGTGCA 3′
	Antisense	5′ GCCACCATGGAGGGCGGATT 3′
ARF5	Sense	5′ ATCTGTTTCACAGTCTGGGACG3′
	Antisense	5′ CCTGCTTGTTGGCAAATACC3′

Supplementary Table S5: Sequences of miR-374a mimics and negative control, miR-374a inhibitors and inhibitors negative control

Gene		Sequence
miR-374a mimics	Sense	5′ UUAUAAUACAACCUGAUAAGUG3′
	Antisense	5′ CUUAUCAGGUUGUAUUAUAAUU3′
Negative control	Sense	5′ UUCUCCGAACGUGUCACGUTT3′
	Antisense	5′ ACGUGACACGUUCGGAGAATT3′
miR-374a inhibitors		5′ CACUUAUCAGGUUGUAUUAUAA3′
Inhibitors negative control		5′ CAGUACUUUUGUGUAGUACAA3′

Supplementary Table S6: SiRNA sequences of CCND1

		Sequence
CCND1-1	Sense	5′ UGGAAUAGCUUCUGGAAUU dTdT 3′
	Antisense	3′ dTdA ACCUUAUCGAAGACCUUAA 5′
CCND1-2	Sense	5′ CAAGCUCAAGUGGAACCUG dTdT 3′
	Antisense	3′dTdT GUUCGAGUUCACCUUGGAC 5′

Supplementary Table S7: Primer sequences for CCND1 3′ UTR

		Sequence
CCND1 3’ UTR	Sense	5′ ATCGCTCGAGACAAAGGAGGCGTCTCGG 3′
	Antisense	5′ ATCGCGGCCGCTAGGAGTGGGACAGGTGGC 3′
